# How to register static occlusion – Correlation of contemporary techniques

**DOI:** 10.1016/j.heliyon.2024.e28130

**Published:** 2024-03-14

**Authors:** Bernhard Wiechens, Phillipp Brockmeyer, Tristan Hampe, Andrea Schubert, Ralf Bürgers, Torsten Wassmann

**Affiliations:** a Department of Prosthodontics and Department of Orthodontics, University Medical Center Göttingen, Göttingen, Germany; b Department of Maxillofacial Surgery, University Medical Center Göttingen, Göttingen, Germany; c Department of Prosthodontics, University Medical Center Göttingen, Göttingen, Germany; d Professor and Head of Department, Department of Prosthodontics, University Medical Center Göttingen, Göttingen, Germany

## Abstract

**Statement of problem:**

A working knowledge of the analytical capacities of contemporary registration methods is essential for prosthetic treatment; however, there is a paucity of studies which coherently investigate the capabilities and limitations of the various diagnostic procedures utilized for prosthetic occlusion.

**Purpose:**

The present prospective clinical study aimed to evaluate the similarities and differences among contemporary registration methods through comparative analysis.

**Material and methods:**

The habitual static occlusion of 19 healthy individuals (14 women; mean age ± standard deviation, 30.8 ± 4.8 years) was analyzed 3 times a day, using shimstock foil, occlusal foil, wax registration, silicone registration, and computerized registration. The procedures were repeated after 14 days. Statistical analyses included all registrations referencing the first measurement point to assess the mean values of antagonistic contacts and the differences between these measurements. Pearson's and Kendall's correlation analyses were performed as part of the coherent mixed logistic regression model, and marginal probabilities were calculated using the registration technique and repeated measurements.

**Results:**

Strong correlations were found among the various registration techniques. The largest effect sizes were observed among the wax, silicone, occlusion foil, and computerized registrations (r = 0.95, *P* < 0.001 to r = 0.62, *P* < 0.001), while the lowest effect sizes were found for shimstock correlations (τ = 0.41, *P* < 0.001 to τ = 0.27, *P* < 0.001). Occlusal changes per maxillary arch were observed referencing the first measurement time with wax registration (*P* < 0.001; 7.4%), shimstock foil (*P* < 0.001; 13.8%), computerized registration (*P* < 0.001; 20.3%), silicone registration (*P* = 0.009; 66.3%), and occlusion foil (*P* < 0.001; 98.8%). Occlusal changes per maxillary tooth were observed from the first incisor (*P* < 0.001; 5.7%) to the third molar (*P* < 0.001; 18.1%).

**Conclusions:**

The results of the present study revealed that there are strong overall correlations among the various contemporary registration techniques. The different affinities of the techniques used to register occlusal changes, however, showed differences in the measurement techniques, which should be neither over- nor underestimated. The differential tendencies of teeth to change should be considered, even if a hypervariable system is assumed.

## Highlights

• This study faces the challenges of coherently assessing dental measurement validity.
• Dental registration methods mostly show high correlations.
• Healthy masticatory systems can be considered hypervariable.
• Healthy occlusion is practically impossible to reproduce.
• Tendency of the antagonism to change increases from anterior to posterior.

## 1 Introduction

The accurate assessment of an individual's static occlusion is a basic requirement for the initial evaluation of the stomatognathic system and for the successful implementation of conservative and restorative interventions [1,2]. A healthy stomatognathic system displays a broad functional range, aiming to avoid repetitive and constant functional patterns to protect against excessive wear and system overload [3]. For decades, however, occlusal analysis has been subject to a paradoxical uncertainty – as there is no gold standard for occlusal registration [1,[4], [5], [6]], it is unknown whether these registrations accurately reflect the high degree of variability within the stomatognathic system or merely reveal the inaccuracy of the measurement techniques themselves [2]. Clinically, this uncertainty has been addressed either using a simplistic view of the stomatognathic system as invariant, whereby the agreement of the repeated measurements obtained during a registration procedure encodes an accurate registration and vice versa [2,7], or using the more physiological approach of presupposing the stomatognathic system as a variable, although contemporary qualitative registration methods have faced the almost impossible task of registering reliably [2,3].

Current studies that followed the latter approach, in which a hypervariant stomatognathic system was presupposed, concluded that even using the most sophisticated digital measurement methods, numerous functional patterns can be found in healthy stomatognathic systems [3]. Consequently, occlusal registration cannot be reproduced *in vivo*, or can only be reliably recorded situationally [3].

Given these limitations, the present study aimed to coherently analyze the registration methods evaluated in previous studies [4,5,[8], [9], [10], [11], [12], [13]], based on the null hypothesis that the various methods of static occlusion registration do not differ significantly from each other. Consequently, the objective of the present study was to provide an overview of the analytical performance of the current registration techniques and recommendations for practitioners in selecting the most appropriate technique.

## 2 Material and methods

**Fig. 1:**
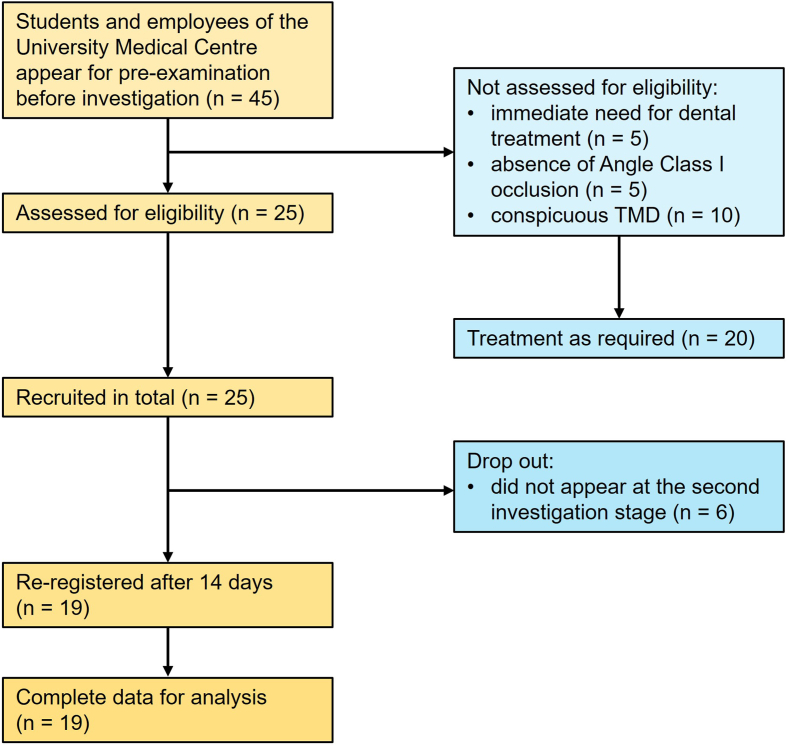
STROBE diagram. Recruitment and analysis process of the present study.

**Fig. 2:**
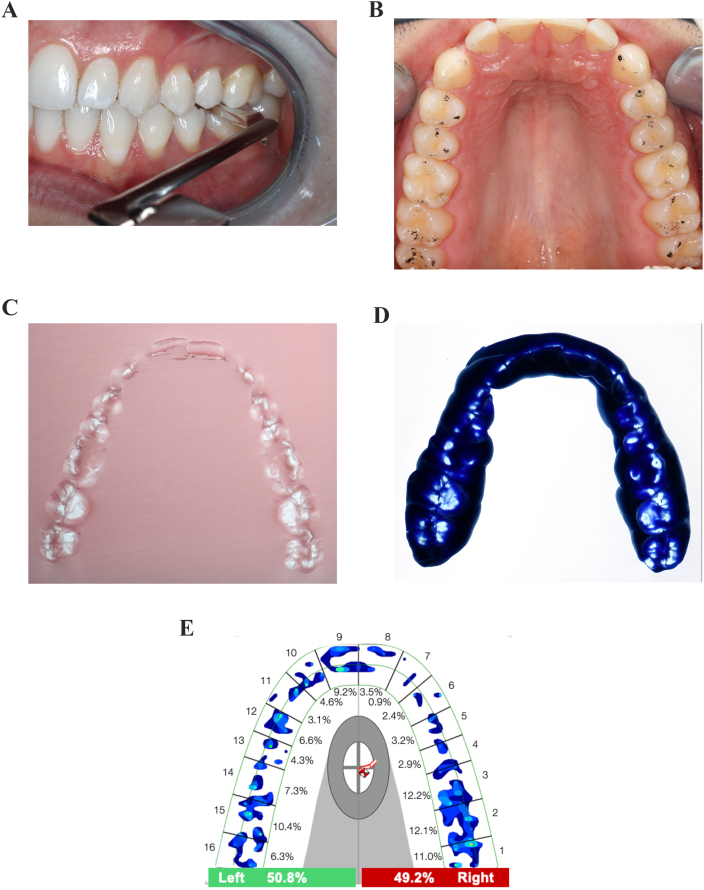
Overview of registration techniques. (A) 8 μm Shimstock foil; (B) black 12 μm occlusion foil; (C) modeling wax; (D) silicone elastomeric impression material; and (E) T-Scan III computerized occlusal analysis.

The protocol of the present study was approved by the Ethics Committee of the University Medical Center (application number 18-9-17) and registered in the National Primary Register (DRKS00029145) approved by the World Health Organization. The present study included 19 healthy adult volunteers (14 female, 5 male), with an Angle class I occlusion plus horizontal and vertical overlaps of 2 ± 1 mm. The cohort for the present study was recruited from patients who participated in a previous investigation [14]. All participants were fully informed about the study procedures and gave their written informed consent to participation and publication of the results. Additionally, the present report complied with the Strengthening of the Reporting of Observational Studies in Epidemiology (STROBE) guidelines for observational studies (Fig. 1). The inclusion criteria were as follows: complete dentition (≥24 teeth); excellent oral hygiene; and the absence of periodontal disease (plaque indices <15%, sulci bleeding indices <10%) [15]. Furthermore, according to the Diagnostic Criteria for Temporomandibular Disorders (DC/TMD) [16], functional abnormalities could be excluded. All clinical examinations were performed by a dentist (B.W.), and static occlusion was recorded using a shimstock foil (Fig. 2A), black occlusal foil (Fig. 2B), wax and silicone (Fig. 2C and D), and computerized (Fig. 2E) occlusal registrations. The participants received detailed instructions for each registration procedure, which were practiced several times before recording the final measurement. For the measurement of static occlusion, the participants were instructed to occlude in the maximum intercuspation position (MIP) [17] on the command “close.” Special instructions were provided to clench the teeth as hard as necessary to ensure that the maxillary and mandibular teeth were in full contact. The occlusion of each participant was recorded using each of the aforementioned methods at 9 a.m., 12 p.m., and 4 p.m. on the day of examination, and again at the same times of day 14 days later. The clinical assessment was performed in an upright sitting position with the head relaxed against the chair's headrest.

Each patient's occlusion was measured using an 8 μm thick shimstock foil (HANEL Shimstock Foil 8μ; Coltène, Switzerland) and a black 12 μm thick occlusal foil (HANEL Occlusion Foil 12μ single-sided; Coltène, Switzerland). The occlusal registration and analysis with the black 12 μm thick occlusal foil were performed as described by Wiechens et al. [14]. The shimstock foil registration was performed using the recommendations described by Harper and Setchell [10], in which each tooth pair (tooth articulating with its paired antagonist in MIP) was evaluated via tensile testing. The absence of a direct contact-point relationship was indicated by pulling the foil out from between the opposing teeth without friction, while pulling out the foil with friction resulted in a regular contact point. If the foil was completely enclosed in the contact area, a strong contact was detected. The registrations were categorized as follows: (−) for absent; (±) for regular; and (+) for strong contact.

Next, wax and silicone occlusal recordings were used to analyze static occlusion. Wax registrations were recorded using modeling wax (HS modeling wax, Henry Schein Inc., USA), while silicone registrations were recorded using an addition silicone elastomeric impression material for bite registration (Registrado X-tra, Voco GmbH, Germany). Considering the material-specific handling of silicone, the participants were asked to achieve MIP on command within 30 s of working time after the application of the unprocessed material on the mandibular dentition. The participants then rested in MIP for 40 s, or until the material was processed. Wax registration was performed using water-warmed wax plates, and due to the rapid solidification of the wax outside of the water bath, the patients were instructed to perform (MIP) immediately after plate placement and to remain there until the temperature reduction allowed for the appropriate solidification of the material. Transillumination and calipers (E110W; Kroeplin GmbH, Germany) were used for the analysis of occlusal recordings. Based on the procedure described by Hützen et al. [18], translucent contact areas with a thickness of ≤20 μm were recorded as contact points, and segmented patterns divided the contact area into several contact points if the defined diameter was not exceeded.

Finally, a computerized occlusal analysis system (T-Scan-III and T-Scan 8.1; Tekscan, Inc., USA) was used for the digital analysis of each patient's static occlusion. The recording and assessment procedures for the computerized registrations were similar to those previously described by Wiechens et al. [14].

Categorical variables were presented as absolute and relative frequencies, while continuous and ordinally scaled variables were presented as means and standard deviations (SDs). The recorded data were categorized for modeling purposes, and for statistical analysis, the first measurement (9 a.m.) on the first day of the study was set as the reference for subsequent measurement time points to examine the correlations among the registration methods.

Considering the findings of Wiechens et al. [14], single-tooth and multiple-teeth (≥2) analyses for full arch evaluation were performed. An increase or decrease of ≥5% in the occlusal contacts between the measurement time points per tooth and arch was considered in the underlying statistical model, and occlusion was coded as constant if the number of contact points on a tooth or arch remained unchanged, taking a <5% threshold into account. The various registration techniques were evaluated using Pearson's (to measure linear relationships) and Kendall's rank (to measure monotonic relationships) correlations. The correlative relationships was presented using r for Pearson's and τ for Kendall's correlations. Pearson's r was interpreted according to Cohen's [19] and Kandall's τ, according to Walker's [20] recommendations. Potential influencing factors that could affect the detection of occlusal changes, such as registration procedures, tooth groups, areas of interest (changes per tooth or arch), and their interaction, were investigated using mixed logistic regression considering the influence of repeated measurements per participant through Holm's method [21]. To illustrate the results, the estimated marginal probabilities for detecting occlusal changes were calculated, taking into account the measurement method and repeated measurements. All analyses were performed by using a statistical programming environment (R v3.5.0; R Core Team 2018, Austria) (α = 0.05 for all statistical tests).

## 3 Results

Table 1 illustrates a comprehensive data set of a study participant completing all measurements.

**Table 1:** Complete data set of a study participant. Time point and daytime code for the measurement day and the respective measurement time. According to registration techniques, scales code for the corresponding scale levels of the measured values. Tooth codes for the respective test tooth of the participant.

Time point	Scale	Registration technique	Day time	Tooth															
				18	17	16	15	14	13	12	11	21	22	23	24	25	26	27	28
**Initial**	ordinal	T-Scan	9 a.m.	9.9	8.3	1.3	0	10.5	1.1	1.3	15.4	5.1	1.3	4.4	7.6	1	11.8	10.8	10.3
		T-Scan	12 p.m.	13.5	8.6	7	1.6	6.7	1.8	3.7	7.4	4.2	1.1	1	2.9	2.9	15.2	12.3	9.8
		T-Scan	16 p.m.	9.7	12.7	8.3	1.3	8.9	1.4	5.4	12	6.2	1.1	0	3.1	1.6	12.1	10.4	5.4
	metric	Silicone (<20 μm)	9 a.m.	9	7	8	5	4	3	1	3	3	1	1	5	5	13	10	15
		Silicone (<20 μm)	12 p.m.	9	7	8	5	4	3	2	3	3	1	1	5	5	12	10	13
		Silicone (<20 μm)	16 p.m.	8	7	8	5	4	3	1	3	3	1	1	5	5	15	10	13
	metric	Wax (<20 μm)	9 a.m.	10	7	7	4	4	3	1	1	1	1	1	4	5	11	10	10
		Wax (<20 μm)	12 p.m.	10	7	7	4	4	3	1	1	1	1	1	4	5	11	10	10
		Wax (<20 μm)	16 p.m.	10	7	7	4	4	3	1	1	1	1	1	4	5	11	10	10
	nominal	Shimstock (8 μm)	9 a.m.	+	+	+	+	+	+	±	–	+	±	+	+	+	+	+	+
		Shimstock (8 μm)	12 p.m.	+	+	+	+	+	+	+	+	–	–	+	+	+	+	+	+
		Shimstock (8 μm)	16 p.m.	+	+	+	+	+	+	–	–	–	–	+	+	+	+	+	+
	ordinal	Occlusal Foil (12 μm)	9 a.m.	3	6	7	6	6	1	1	2	1	1	1	4	4	7	8	9
		Occlusal Foil (12 μm)	12 p.m.	4	6	6	7	6	1	1	2	1	1	1	4	4	5	8	9
		Occlusal Foil (12 μm)	16 p.m.	5	6	6	5	5	1	1	1	1	1	1	5	6	7	8	9
**After 14 days**	ordinal	T-Scan	9 a.m.	6	10.2	6.8	4.8	6.6	3.5	4.6	9	3.4	1.1	2.4	3.1	3.1	12.5	12.3	10.7
		T-Scan	12 p.m.	6.8	10.3	8	4.3	6.4	2.5	3.6	7.5	4	1.2	0.9	2.4	7	12	14.7	10
		T-Scan	16 p.m.	10.8	10.5	8	3.8	5.6	1.8	3.9	8.7	4.7	1.4	1.8	2	5.8	12.8	14.8	5.7
	metric	Silicone (<20 μm)	9 a.m.	8	7	8	5	4	2	1	3	3	1	1	5	5	12	11	13
		Silicone (<20 μm)	12 p.m.	10	7	8	5	5	2	1	3	3	1	1	5	5	12	11	13
		Silicone (<20 μm)	16 p.m.	10	7	8	5	4	3	1	3	3	1	1	5	5	12	11	13
	metric	Wax (<20 μm)	9 a.m.	10	7	7	4	4	3	1	1	1	1	1	4	4	11	10	10
		Wax (<20 μm)	12 p.m.	10	7	7	4	4	3	1	1	1	1	1	4	4	11	10	10
		Wax (<20 μm)	16 p.m.	10	7	7	4	4	3	1	1	1	1	1	4	4	11	10	10
	nominal	Shimstock (8 μm)	9 a.m.	+	+	+	+	+	+	–	–	–	–	+	+	+	+	+	+
		Shimstock (8 μm)	12 p.m.	+	+	+	+	+	+	–	–	–	–	+	+	+	+	+	+
		Shimstock (8 μm)	16 p.m.	+	+	+	+	+	+	–	–	–	–	+	+	+	+	+	+
	ordinal	Occlusal Foil (12 μm)	9 a.m.	5	5	6	6	6	2	1	1	1	1	1	5	7	6	6	6
		Occlusal Foil (12 μm)	12 p.m.	6	6	6	5	6	1	1	2	1	0	1	5	7	8	6	4
		Occlusal Foil (12 μm)	16 p.m.	4	6	6	4	6	2	1	1	1	0	1	5	6	8	8	5

Table 2 shows the mean absolute counts of occlusal contacts per tooth for the wax, silicone, and occlusal foil registrations, while T-Scan registration is shown as a percentage of the applied force, and shimstock registration as the absolute number of each finding. Silicone registration exhibited the most static contact points per tooth (4.5 ± 3.2, mean ± SD), while occlusal foil registration registered the least (3.7 ± 2.7, mean ± SD). As the scaling of the variables differed, no direct contact point comparison could be performed for the T-Scan registrations [14].

**Table 2:** Results of static occlusion measurements. Measured values presented as contacts per tooth for wax, silicone, and occlusal foils, percentage of total force load for T-Scan III, and descriptive values for shimstock foil.

Registration technique	Mean ± standard deviation (SD)	Value	Dimension
Wax	mean ± SD	4.1 ± 2.9	contacts per tooth
Occlusal foil	mean ± SD	3.7 ± 2.7	
Silicone	mean ± SD	4.5 ± 3.2	
Shimstock	minus	128 (8.0%)	total findings
	plus/minus	118 (7.3%)	
	plus	1362 (84.7%)	
T-Scan	mean ± SD	6.8 ± 5	percentage of total force load per tooth

**Fig. 3:**
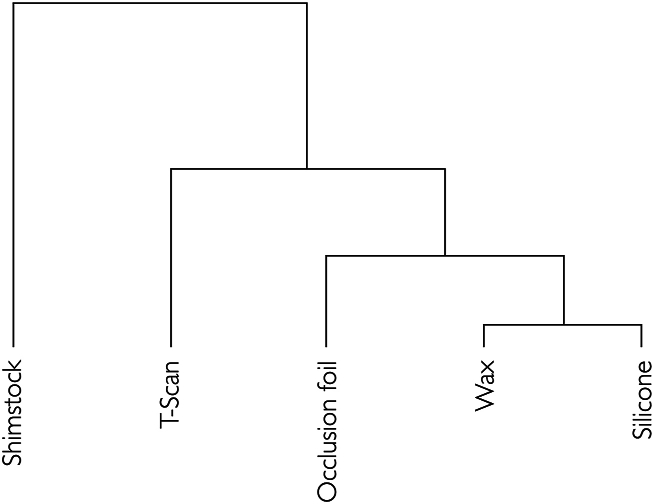
Hierarchic clustering of measurements. Visualization of correlative relationships as a hierarchical cluster. Small clusters indicate strong correlations, large clusters indicate weak correlations. Occlusion foil and T-scan show a strong correlation, silicone and wax show a very strong correlation. Shimstock forms a separate group because of nominal scaled variables.

The various registration methods correlated with the detection of possible occlusal changes between the measurements (Table 3, Fig. 3) as follows: strong correlations were found between wax, silicone, occlusion foil, and T-Scan registrations (r = 0.62, *P* < 0.001 to r = 0.95, *P* < 0.001), and shimstock foil registration showed moderate correlations with the other techniques (τ = 0.27–0.42).

**Table 3:** Correlation of various registration techniques. Pearson's and Kendall's correlation coefficients presented as r- and τ-values. Adj.*P* indicates Bonferroni-Holm adjusted *P*-values. Effect size interpretation based on Cohen's and Walker’ recommendations.

**Registration technique**	**Correlation coefficient**	***P-*value**	***Adj.P***	**Effect size**
Shimstock/T-Scan	τ = 0.27	<0.001	<0.001	moderate
Wax/Shimstock	τ = 0.40	<0.001	<0.001	
Silicone/Shimstock	τ = 0.41	<0.001	<0.001	
Occlusion foil/Shimstock	τ = 0.42	<0.001	<0.001	
Occlusion foil/T-Scan	r = 0.62	<0.001	<0.001	large
Wax/T-Scan	r = 0.65	<0.001	<0.001	
Silicone/T-Scan	r = 0.68	<0.001	<0.001	
Wax/Occlusion foil	r = 0.80	<0.001	<0.001	
Occlusion foil/Silicone	r = 0.82	<0.001	<0.001	
Wax/Silicone	r = 0.95	<0.001	<0.001

Table 4 shows the probabilities calculated for each method for detecting occlusal changes per tooth, according to which, the probability of detecting an occlusal change in subsequent measurements using occlusal foil was 41.6%, whereas the marginal probabilities of detecting an occlusal change with shimstock and wax registration were only 3.2% and 3.8%, respectively. The probability of detecting an occlusal change with silicone was 18.4% and one fifth at 7.5% for T-Scan registration.

**Table 4:** Marginal probabilities per technique for registering occlusal changes for any tooth. Probabilities per tooth.

Registration technique	**Probability**	**Confidence interval**	***P-*value**	**Significance**
Wax	3.8%	(2.7–5.2)	<0.001	***
Occlusion foil	41.6%	(37–46.4)	<0.001	***
Silicone	18.4%	(15.3–21.8)	<0.001	***
Shimstock	3.2%	(2.3–4.6)	<0.001	***
T-Scan	7.5%	(5.8–9.6)	0.001	**

To observe occlusal changes per jaw, at ≥ 2 teeth had to show a significant occlusal change. The relationships of the marginal probabilities calculated among all tested registration techniques were similar to those of single-tooth observations, although their absolute values were significantly higher overall (Table 5). Therefore, for registrations using occlusal foils, a marginal probability of 98.8% could be assumed for occlusal changes per jaw between measurements, indicating more than a duplication of the probability. Similarly, the probability of wax doubled, that of silicone increased by more than threefold, and that of shimstock increased by more than fourfold.

**Table 5:** Marginal probabilities per technique for registering occlusal change per jaw (≥2 teeth with occlusal changes). Probabilities per jaw.

Registration technique	Probability	Confidence interval	*P-*value	Significance
Wax	7.4%	(3.3–15.9)	<0.001	***
Occlusion foil	98.8%	(91.7–99.8)	<0.001	***
Silicone	66.3%	(54.1–76.7)	0.009	**
Shimstock	13.8%	(7.6–23.8)	<0.001	***
T-Scan	20.3%	(12.4–31.4)	<0.001	***

**Fig. 4:**
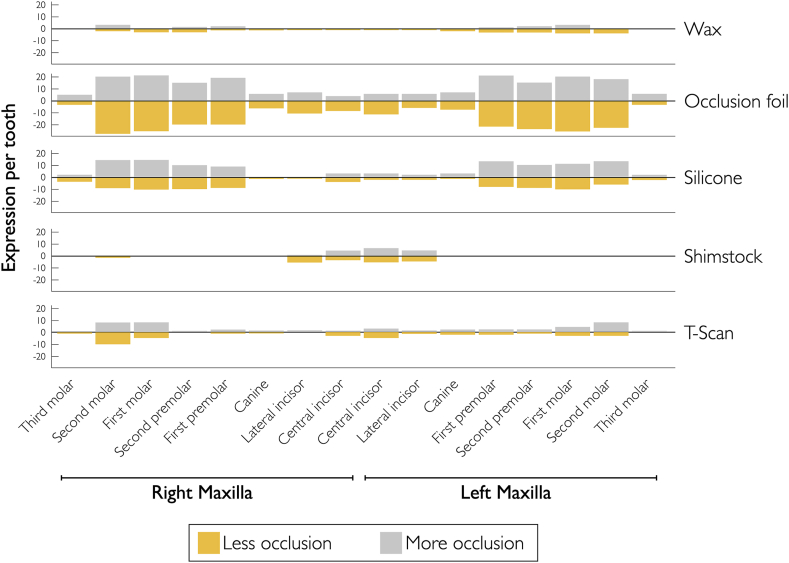
Occlusal changes. Expression of registered changes of overall measurements per procedure and tooth in percentages.

**Fig. 5:**
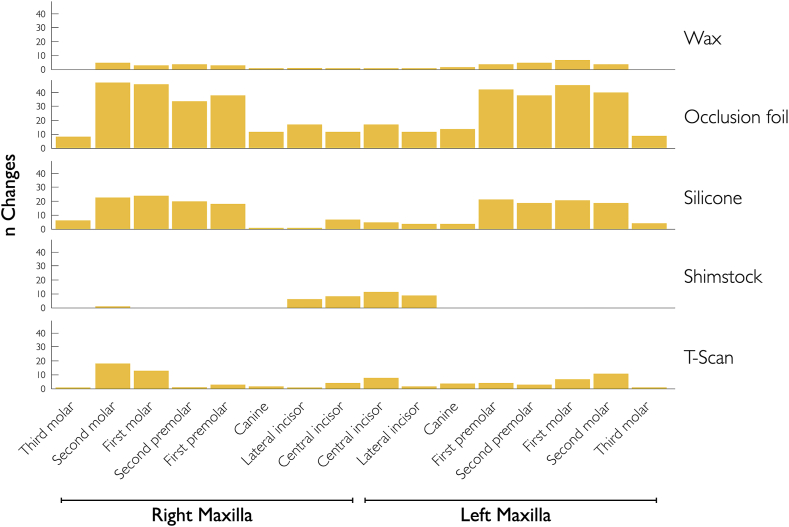
Capacity for change. Absolute expression of degree of change (n changes) by registration method and tooth in percentages.

Table 6 presents the marginal probabilities of detecting occlusal changes in subsequent registrations for all procedures, based on the type of teeth. Probabilities increased from anterior to posterior teeth, with incisors having a probability of 5.7%, and molars ranging from 15.1 to 18.1%. Fig. 4, Fig. 5 provide a comprehensive overview of the possibility and extent of change in occlusion in subsequent measurements. Logistic regression accounted for the potential inhomogeneity among the study population.

**Table 6:** Marginal probabilities of registering occlusal change on specific tooth. Probabilities of teeth.

Tooth	Probability	Confidence interval	*P*-value	Significance
1	5.7%	(4.5–7.3)	<0.001	***
2	6.7%	(5.3–8.4)	<0.001	***
3	6.4%	(5.1–8.1)	<0.001	***
4	12.3%	(10.2–14.8)	<0.001	***
5	11.6%	(9.5–14.1)	<0.001	***
6	14.8%	(12.4–17.6)	<0.001	***
7	15.1%	(12.6–17.9)	<0.001	***
8	18.1%	(12.6–25.3)	<0.001	***

## 4 Discussion

The results of the present study revealed that different techniques for static occlusion registration showed an overall acceptable correlation regarding the measurement of hypothesized occlusal changes. However, they differed considerably in terms of information value and content, which emphasises the review results recently reported by Qadeer et al. [22]. The null hypothesis "Various methods of static occlusion registration do not differ significantly from each other " was therefore rejected.

The sample population of the present study consisted predominantly of young healthy female adults with Angle Class I occlusions, sagittal and vertical overlaps of 2 ± 1 mm and absence of functional pathologies. A population bias due to dysgnathia or dysfunction could therefore be excluded.

Regarding the design of the present study, we note that although there are numerous articles on different occlusal registration methods, there is a lack of studies focusing on the totality of common registration methods in prospective longitudinal observations with multiple measurement points, one study population, and a coherent data analysis using logistic regression modeling. With hindsight, this may be explained by the difficulty of modeling variables in a coherent model that incorporates nominal, ordinal and metric scales. However, the comprehensive statistical framework of the present study made it possible to adequately address this ambiguity. Not least, this is confirmed by the results of the existing literature on corresponding methods [1,22].

Based on the results presented, it furthermore could be assumed that the cause of variable measurement results, was attributable to a considerable extent through the hypervariability of the masticatory system [2,3,18]. Changes in occlusal patterns recorded with different techniques showed lower (wax) to higher (occlusal foil) marginal probabilities, but globally indicated the trend of occlusal change consistently in all methods, which in several cases allowed high correlations to be observed. However, despite the overall strong and significant correlation between the registration techniques, apart from Shimstock registration, the strongest correlation was found for wax and silicone. This correlation pair clearly illustrated the nature of this investigation, as the detection of contact point patterns was initially found to be highly consistent. However, according to the literature, the reliability of wax registrations is low, not least because of the material-specific properties [1,22]. This could be concluded from the available results of the calculated change probabilities for each method, with wax (7.4%) and occlusion foil (98.9%) defining the lower and upper limits respectively. Against the background of specificity and sensitivity weaknesses, which have been strongly criticised in the literature for both methods [1,13,22], a relativization had to be made here as well. The fundamental difficulty in determining the most suitable means of registration is the absence of a specific gold standard and again the hypervariability of the masticatory system under investigation [2,3,18]. However, recent studies have shown that electronic measurement methods are largely able to overcome these criticized specificity flaws [13,22], which has shifted the interpretation of the calculated marginal probabilities to the focus of the T-scan and silicone registration. Concerning the question which analogue registration technique to prefer, the literature suggests that silicone registration is more reliable, as it shows negligible occlusal resistance in the initial phase of curing and high strength and dimensional stability in the processed state, which could also be emphasized in view of the presented study results [9,12]. In comparison to the other methods, silicone and T-scan registration also showed a rather moderate affinity to indicate excessive occlusal changes. This should be seen as an advantage, since in addition to the already described high correlations with other registration techniques, either very high (occlusal foil) or very low probabilities (wax registration) for changes were observed. Ultimately, silicone has an advantage in the evaluation of the different techniques mainly because of its accuracy, which enables reliable occlusal assessment [9]. Other methods generally cannot offer this accuracy and reliability, which is due to the low torsional stability, temperature susceptibility and bite blocking of wax [8], the unreliable registration of occlusal foils in a moist environment [1,4], or the frequently described poor marking behavior [13].

Furthermore, differences were observed in the applicability of the various techniques over the entire dental arch. Due to the relatively limited information content of Shimstock foils and the multiple occlusion cycles during the dental arch registration, allowing the assumption of a constantly slightly changed bite position, Shimstock foils had to be regarded as unsuitable [1,4]. The other registration techniques, on the other hand, showed clearer but nevertheless different increases in the detected marginal probabilities in this context, which can be considered comprehensible again in terms of hypervariance [3]. Particular attention was paid to the T-scan registration, which was mid-range regarding correlation and detection of occlusal changes, but did not show any significant limitations in the overall arch observation or generation of false-positive registrations [13]. At the same time, the computerized registration technique provided significantly more information than all other methods [14,22]. It should be noted, however, that the T-scan registrations were performed through a sensor with a diameter of 100 μm, leading to significantly more bite blocking during registration than, for example, occlusal foil with a diameter of 12 μm [23].

Apart from the techniques investigated, the results of occlusal variability could support the modern approach of Kordaß [3] and Türp [2], considering occlusion as an additional variable in a hypervariable system, prescribing self-protection in addition to its actual function [3]. This was particularly apparent by observing that anterior teeth changed less than posterior teeth, as they account for a relatively small proportion of overall masticatory performance. Since incisors are exposed to relatively lower loads than molars due to their primary tearing function in masticatory tasks, repetitive loads may be considered less influential on occlusal wear [11,[24], [25], [26]]. This could explain the significant probability gradient observed, which ranged from 5.7% on central incisors to 15.7% on secondary molars [[24], [25], [26]].

In conclusion, all static occlusion registration methods assessed in the present study have limitations and are therefore not suitable for every clinical task. However, it should be noted that silicone was superior due to its static accuracy and T-scan due to its significantly higher information content [22].

Furthermore, it should be sensitized to the fact that static occlusion itself can only be regarded as a snapshot, which raises the question of whether the highest precision of a singular functional state is ultimately relevant at all. Rather, the acquisition of multi-bite registrations, which are possible by means of computer-aided registrations, might be more appropriate [22]. This would allow digitally planned restorations to be superimposed on multiple occlusion scenarios in order to achieve an interference-free and functional design. In addition, digital systems are also suitable for evaluating the functional status of the masticatory system via opening and closing velocities of disclusion an occlusion, resulting in considerable advantages for longitudinal observations of function-motivated treatments, which will define the future but also more cost-intensive standard.

The limitations of the present study include the fact that all registration techniques were investigated in a healthy hypervariable system. Furthermore, it can be assumed that the investigated techniques directly influence the occlusion due to their material-specific properties, so that a bias can be assumed for all measurements [23]. In addition, due to the different measurement times and methods, a different occlusal intensity can be assumed for each subject, which was only controlled narratively and not quantitatively via surface electromyography. Finally, a large number of alternative registration procedures such as articulating papers, occlusion sprays, photo occlusion and occlusion sonography should be mentioned, which were not evaluated in the present study, so that the study cannot be regarded as complete coverage of all possible registration procedures.

## 5 Conclusions

Based on the results of the present study, the following conclusions may be drawn.

1. Overall, good correlations among the common registration techniques can be assumed.
2. The information content and accuracy of the techniques evaluated vary significantly, and their selection should be situation-dependent.
3. Every registration technique must be considered as a snapshot from a hypervariable system, irrespective of the method used.

## 6 Clinical implications

When evaluating data gathered under clinical conditions, the methods tested for recording static occlusion differed significantly in their analytic results, despite a high correlation. Based on those results, silicone registration should be favored, due to its high clinical reliability, while computerized registration should be favored because of its capabilities for quantitative analysis providing additional information related to the occlusal process.

## Ethical statement

The ethics committee of the University Medical Centre Göttingen has approved this study under the application number (18-9-17), that is also registered in the corresponding national primary register (DRKS00029145).

## Consent to participate

All participants were fully informed about the study procedures and gave their written informed consent to participate.

## Consent for publication

All participants gave their written informed consent to the publication of the results.

## Funding

This research did not receive any specific grant from funding agencies in the public, commercial, or not-for-profit sectors.

## Data availability statement

Data included in article/supp. material.

## CRediT authorship contribution statement

**Bernhard Wiechens:** Writing – review & editing, Writing – original draft, Methodology, Investigation, Formal analysis, Conceptualization. **Phillipp Brockmeyer:** Writing – review & editing, Investigation, Formal analysis. **Tristan Hampe:** Writing – review & editing, Investigation, Formal analysis. **Andrea Schubert:** Writing – review & editing, Investigation, Formal analysis. **Ralf Bürgers:** Writing – review & editing, Methodology, Investigation, Formal analysis, Conceptualization. **Torsten Wassmann:** Writing – original draft, Methodology, Investigation, Formal analysis, Conceptualization.

## Declaration of competing interest

The authors declare that they have no known competing financial interests or personal relationships that could have appeared to influence the work reported in this paper.
